# Correction: Development of hollow ferrogadolinium nanonetworks for dual-modal MRI guided cancer chemotherapy

**DOI:** 10.1039/c9ra90017a

**Published:** 2019-03-05

**Authors:** Ting Tang, Xiao Sun, Xuedong Xu, Yifeng Bian, Xiaojie Ma, Ning Chen

**Affiliations:** Jiangsu Key Laboratory of Oral Diseases, Nanjing Medical University Nanjing 210029 China dentistchenning@126.com; Department of Dental Implantology, Hefei Stomatology Hospital, Clinical School of Anhui Medical University Hefei 230001 China; Department of Chemical and Biomolecular Engineering, National University of Singapore Singapore 117585 Singapore xiaosun8000@163.com; China Shijiazhuang Pharmaceutical Group Co., Ltd. Shijiazhuang 050038 China

## Abstract

Correction for ‘Development of hollow ferrogadolinium nanonetworks for dual-modal MRI guided cancer chemotherapy’ by Ting Tang *et al.*, *RSC Adv.*, 2019, **9**, 2559–2566.

The authors regret that Scheme 1 in the original article included some incorrect data under the cancer chemotherapy part of the scheme. The correct version of Scheme 1 is presented below.

**Scheme 1:**
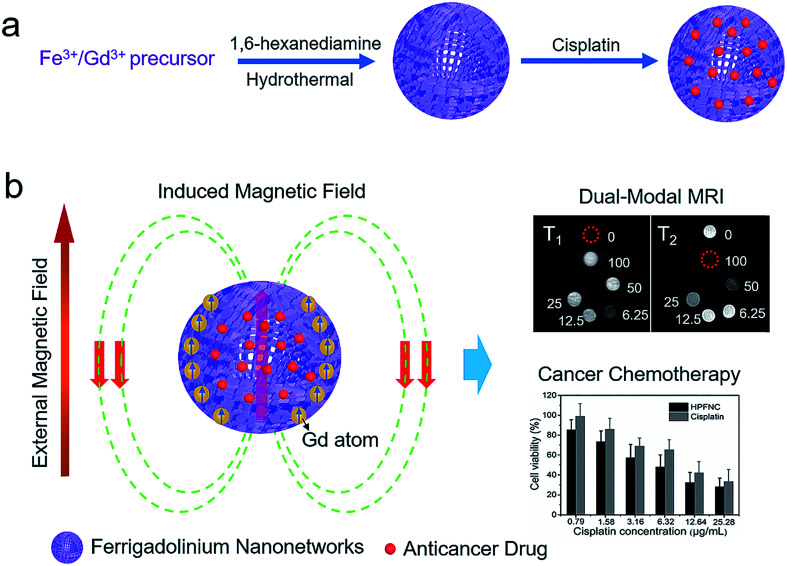


The Royal Society of Chemistry apologises for these errors and any consequent inconvenience to authors and readers.

## Supplementary Material

